# The Synergy of Machine Learning and Epidemiology in Addressing Carbapenem Resistance: A Comprehensive Review

**DOI:** 10.3390/antibiotics13100996

**Published:** 2024-10-21

**Authors:** Aikaterini Sakagianni, Christina Koufopoulou, Petros Koufopoulos, Georgios Feretzakis, Dimitris Kalles, Evgenia Paxinou, Pavlos Myrianthefs, Vassilios S. Verykios

**Affiliations:** 1 Intensive Care Unit, Sismanogleio General Hospital, 15126 Marousi, Greece; sakagianni@sismanoglio.gr; 2 Anesthesiology Department, Aretaieio Hospital, National and Kapodistrian University of Athens, 11528 Athens, Greece; ckoufopoulou@uoa.gr; 3 Internal Medicine Department, Sismanogleio General Hospital, 15126 Marousi, Greece; smd1600078@uoa.gr; 4 School of Science and Technology, Hellenic Open University, 26335 Patras, Greece; georgios.feretzakis@ac.eap.gr (G.F.); kalles@eap.gr (D.K.); paxinou.evgenia@ac.eap.gr (E.P.); 5 Faculty of Nursing, School of Health Sciences, National and Kapodistrian University of Athens, 11527 Athens, Greece; pmiriant@nurs.uoa.gr

**Keywords:** carbapenem resistance, machine learning, epidemiology, antimicrobial resistance, predictive modeling, public health

## Abstract

Background/Objectives: Carbapenem resistance poses a significant threat to public health by undermining the efficacy of one of the last lines of antibiotic defense. Addressing this challenge requires innovative approaches that can enhance our understanding and ability to combat resistant pathogens. This review aims to explore the integration of machine learning (ML) and epidemiological approaches to understand, predict, and combat carbapenem-resistant pathogens. It examines how leveraging large datasets and advanced computational techniques can identify patterns, predict outbreaks, and inform targeted intervention strategies. Methods: The review synthesizes current knowledge on the mechanisms of carbapenem resistance, highlights the strengths and limitations of traditional epidemiological methods, and evaluates the transformative potential of ML. Real-world applications and case studies are used to demonstrate the practical benefits of combining ML and epidemiology. Technical and ethical challenges, such as data quality, model interpretability, and biases, are also addressed, with recommendations provided for overcoming these obstacles. Results: By integrating ML with epidemiological analysis, significant improvements can be made in predictive accuracy, identifying novel patterns in disease transmission, and designing effective public health interventions. Case studies illustrate the benefits of interdisciplinary collaboration in tackling carbapenem resistance, though challenges such as model interpretability and data biases must be managed. Conclusions: The combination of ML and epidemiology holds great promise for enhancing our capacity to predict and prevent carbapenem-resistant infections. Future research should focus on overcoming technical and ethical challenges to fully realize the potential of these approaches. Interdisciplinary collaboration is key to developing sustainable strategies to combat antimicrobial resistance (AMR), ultimately improving patient outcomes and safeguarding public health.

## 1. Introduction

Antimicrobial resistance (AMR) represents a critical and escalating threat to global health, with significant implications for the treatment and prevention of infectious diseases. The rise of AMR is primarily driven by the misuse and overuse of antimicrobials in human medicine, agriculture, and veterinary practices, leading to the emergence of drug-resistant pathogens that are increasingly difficult to treat with existing medications. According to the World Health Organization (WHO), AMR is among the top-ten global public health threats facing humanity. In 2019, bacterial AMR directly caused 1.27 million deaths and contributed to 4.95 million deaths worldwide [1].

The impact of AMR extends beyond health, affecting economic stability and development. Projections indicate that if no effective measures are taken, AMR could lead to 10 million deaths annually by 2050 and could cost the global economy up to USD 100 trillion due to increased healthcare costs, loss of productivity, and other factors [2]. High-income countries are experiencing significant issues with healthcare-associated infections caused by resistant bacteria, such as methicillin-resistant *Staphylococcus aureus* (MRSA), while low- and middle-income countries bear a disproportionate burden of resistant infections due to weaker healthcare infrastructure and surveillance systems [3]. In response to this global crisis, coordinated international efforts are crucial. The WHO emphasizes a One Health approach, integrating actions across human health, animal health, and environmental sectors to combat AMR comprehensively [4]. Strengthening antimicrobial stewardship programs, enhancing infection prevention and control measures, and investing in the development of new antibiotics, vaccines, and diagnostics are pivotal strategies recommended by global health authorities to address AMR effectively [5,6].

While AMR spans a wide range of antimicrobial drugs and pathogens, carbapenem resistance (CR) is particularly alarming. Carbapenems are considered one of the last lines of defense against multidrug-resistant bacterial infections, making CR a significant clinical challenge. The emergence of carbapenem-resistant organisms threatens the effectiveness of this vital class of antibiotics, especially in treating severe hospital-acquired infections [7]. The motivation behind this review is to explore the intersection of machine learning (ML) and epidemiology in addressing CR. The growing complexity and volume of AMR data, combined with the increasing need for rapid predictions, present an opportunity to leverage ML techniques in enhancing the surveillance, prediction, and control of carbapenem-resistant infections (CRIs). 

This review aims to synthesize the current state of research on the use of ML in AMR prediction, particularly focusing on CR, and to propose future research directions that combine these approaches. The review’s relevance lies in its potential to inform public health policies and enhance clinical decision-making in managing CRIs.

The key contributions of this review include the following:

1. Comprehensive overview of synergy. A detailed analysis of how ML and epidemiological methods can complement each other in addressing carbapenem resistance is provided.
2. Identification of gaps in traditional approaches. The review outlines the limitations of traditional epidemiological methods in capturing the complexity of resistance mechanisms and transmission patterns and discusses how ML can fill these gaps.
3. Evaluation of ML applications. It examines the current state of ML applications in antimicrobial resistance, particularly in predicting CR, and the potential effectiveness of these models in clinical and public health settings.
4. Proposals for future research. The review identifies key areas for future research, including the need for more robust data integration, model validation, and the development of real-time surveillance systems.
5. Clinical and public health implications. The review emphasizes the clinical and public health benefits of integrating ML and epidemiology to improve predictions, patient outcomes, and intervention strategies.

The remainder of this paper is organized as follows: Section 2 discusses the epidemiology and mechanisms of CR, including the clinical and public health implications. Section 3 delves into the traditional epidemiological approaches used in studying AMR, highlighting their strengths and limitations. Section 4 focuses on the potential of ML to enhance CR predictions, discussing existing models and applications. Section 5 explores how ML and epidemiological methods can be integrated to provide a comprehensive approach to tackling carbapenem resistance. Section 6 outlines future research directions and recommendations for improving AMR surveillance and prediction systems. Finally, Section 7 concludes the review by summarizing key insights and contributions.

## 2. Specific Focus on Carbapenem Resistance

AMR presents a wide array of challenges, but resistance to carbapenem antibiotics is particularly concerning due to their role as the last effective treatment option for many bacterial infections. The emergence and rapid spread of CR in organisms, such as *Klebsiella pneumoniae* and *Pseudomonas aeruginosa*, exemplifies the severity of the AMR crisis. This specific form of resistance underscores the broader challenge posed by AMR and highlights the urgent need for improved surveillance, prevention, and treatment strategies.

Carbapenem-resistant organisms often exhibit complex resistance mechanisms that make infections difficult to treat and control. Therefore, understanding these mechanisms is essential for developing targeted strategies to combat CR on a global scale [7].

### 2.1. Mechanisms of Resistance

Carbapenem resistance arises through several mechanisms, primarily involving enzymatic degradation, efflux pumps, and porin mutations [8,9]. One of the most critical mechanisms is the production of carbapenemases, a group of enzymes that can hydrolyze carbapenems and other β-lactams, rendering these potent antibiotics ineffective. Notable carbapenemases include KPC (*Klebsiella pneumoniae* carbapenemase), NDM (New Delhi metallo-β-lactamase), VIM (Verona integron-encoded metallo-β-lactamase), and OXA-48 (Oxacillinase-48) [7,8]. These enzymes differ in their genetic origins and geographical distribution, but they all confer high levels of resistance, making infections difficult to treat [9]. The widespread dissemination of these enzymes is largely driven by plasmids, which facilitate the transfer of resistance genes across different bacterial species, thus exacerbating the problem of multidrug resistance [8,9].

Furthermore, efflux pumps are able to actively extrude a wide range of different antibiotics out of the bacterial cells, being one of the most important causes of resistance to carbapenems. In general, this decreases the intracellular drug content to subtherapeutic levels. This not only leads to reduced efficacy of carbapenems but also ensures that bacteria can survive even in environments of high antibiotic pressure. Overexpression of efflux pumps can be driven by genetic changes, or an alteration in regulation, often through the influence of the presence of antibiotics. This underscores the dynamic nature of bacterial adaptation [10].

Besides enzymatic degradation and active efflux mechanisms, the third important mechanism of resistance is changes in the permeability of bacterial cell membranes. It most frequently occurs in the form of mutations in the porin proteins, leading to decreased expression or a complete loss of specific porin channels, acting as portals for antibiotics to cross the bacterial cell membrane. These mutations result in a reduced uptake of carbapenems, preventing the antibiotics from reaching their intracellular targets, such as penicillin-binding proteins [11]. The combined effect of porin loss and cabapenemase production can result in extremely high levels of resistance, posing a significant challenge to treatment [12].

Resistance mechanisms are further complicated by the ability of bacteria to share resistance genes through horizontal gene transfer (HGT). This process allows for the rapid spread of resistance determinants between different bacteria, even across species and genera. HGT occurs through various means, including transformation, transduction, and conjugation, with conjugative plasmids being particularly important in the spread of carbapenem resistance [13]. The global spread of carbapenemase-producing Enterobacteriaceae (CPE) is a testament to the role of HGT in the dissemination of resistance, making it a critical factor in the ongoing battle against antibiotic resistance [7].

The combination of the above resistance mechanisms creates a complex challenge in the control of carbapenem-resistant infections (CRIs) [14]. A multifaceted approach involving new antibiotic development, use of combination therapy, and strict infection control practices is required to limit the dissemination of the resistant bacterial strains. In addition, ongoing surveillance and research on the molecular mechanisms of resistance maintain an edge in this continuing battle [12].

### 2.2. Epidemiology and Incidence of Carbapenem-Resistant Organisms

Resistance to carbapenem has escalated into a health problem worldwide, especially during the last decade. The most threatening ones are the carbapenem-resistant Enterobacteriaceae (CRE), carrying the most concerning ability to cause severe and frequently untreatable infections. The presence of these bacteria has been documented in multiple geographical areas, with strikingly high rates in countries that face specific problems related to poor antibiotic stewardship practices, as well as where overuse of antibiotics occurs both in healthcare and agriculture [15,16].

However, the effects of the emergence and spread of CRE have varied among different European countries. For example, in countries such as Greece and Italy, challenges related to CRE have been especially prominent [16]. The combination of factors, such as heavy antibiotic consumption, high rates of hospital-acquired infections, and poor infection control measures, has contributed to the wide dissemination of this type of resistance, with the majority of outbreaks occurring in healthcare environments [17]. Recent studies showed that hospital-acquired infections in Greece account for approximately 316,000 hospital bed days and EUR 73 million in costs due to resistant pathogens, particularly *Klebsiella pneumoniae* and *Escherichia coli*, with over 50% of *K. pneumoniae* isolates being carbapenem-resistant [18].

It is no different in Asia, as high levels of carbapenem resistance have been reported from a number of countries, such as India and China, where antibiotics are freely available without prescription, healthcare facilities are possibly limited or under-resourced, and there is massive use of antibiotics in agriculture and aquaculture. The carbapenemase-producing organisms (CPOs) have presented tremendous challenges to public health. In India, there have been reports of a high prevalence of CPOs, particularly those carrying the New Delhi metallo-β-lactamase (NDM) gene, in both community-acquired and nosocomial infections [15]. For instance, a study reported that 90.3% of carbapenem-resistant bacteria were carbapenemase producers, with NDM-1 being the most dominant at 47% [19]. Equally burdened by a huge population and significant infectious disease load, China has likewise reported increasing incidences of CRE. Between 2012 and 2016, 85.7% of CRE strains in China carried carbapenemase genes, with KPC being predominant in *K. pneumoniae*, and NDM in *E. coli* and *Enterobacter cloacae*. Moreover, the resistance rates of *K. pneumoniae* to meropenem in China have reached 24.2%, underscoring the severity of the problem [19].

The United States remains no exception to the impending threat of CR pathogens. Reports of CRE infections are increasing all over the country, posing significant threats to public health. The CDC has defined CRE as an urgent threat, and there has been much concern expressed over the potential to spread in healthcare settings. More than 2.8 million antibiotic-resistant infections occur in the U.S. each year, resulting in over 35,000 deaths. CRE, in particular, has been responsible for 13,100 cases and 1100 deaths annually [20]. The outbreaks of CRE in the U.S. healthcare facilities have been associated with high morbidity and mortality, pointing to the need for an improved infection control practice and antibiotic stewardship [20].

Besides CRE, other CR pathogens include *Pseudomonas aeruginosa* and *Acinetobacter baumannii*. These are part of the problem and considerably affect the outcomes of treatment, the prevalence of which has increased with time. Community-acquired pneumonia due to CR *A. baumannii* (CRAB) is also rampant in healthcare settings, especially in the ICUs, with resistance rates described at over 30–90% in regions such as Asia, Eastern Europe, and Latin America [21]. 

Even though it varies across different regions, CR *P. aeruginosa* (CR-PA) shows similar alarming resistance rates worldwide [22]. In the United States, CR-PA is a significant healthcare-associated pathogen, responsible for 10–30% of *P. aeruginosa* isolates. A study highlighted that carbapenemase-producing *P. aeruginosa* is frequently found in ventilator-associated pneumonia (VAP) and catheter-related urinary tract infections, contributing to longer hospital stays and higher mortality rates [22]. Both these non-fermenting CR pathogens, including *A. baumannii*, lead to increased healthcare burdens, accounting for over 80% of CR cases in a five-year U.S.-based study [23]. 

A systematic review of carbapenem resistance in animals, foods, and the environment on the African continent further emphasized the widespread nature of this issue. The review found a pooled prevalence of 19.1% across animal, environmental, and food ecosystems, highlighting *Escherichia* spp. (53.5%), *Klebsiella* spp. (35.4%), and *Pseudomonas* spp. (15.7%) as the predominant CRB species. The most common carbapenemase genes reported were from the blaOXA (52.4%) and blaNDM (40.5%) families. These findings suggest that animal–environment–food ecosystems play a significant role as reservoirs for CRE and other carbapenem-resistant bacteria, further driving their dissemination into human populations [24].

This global and multisectoral spread of carbapenem resistance highlights the urgent need for enhanced surveillance, infection control, and new antibiotics targeting both fermenting and non-fermenting CR pathogens, as well as stricter antibiotic stewardship practices across all sectors.

### 2.3. Clinical Implications

Carbapenem-resistant infections, especially those caused by CPOs, such as *Klebsiella pneumoniae*, have significant clinical relevance due to their association with higher mortality rates. A study conducted in Italian hospitals between 2010 and 2013 reported a 14-day mortality rate of 34.1% among patients with KPC-producing *K. pneumoniae* (KPC-Kp) infections. Risk factors associated with this high mortality include bloodstream infections (BSIs), septic shock, chronic renal failure, and the use of colistin-resistant isolates. Inadequate empirical therapy further elevates these risks [25]. A meta-analysis of worldwide studies on CRE infections reported that 26–44% of deaths in patients with CRIs were directly attributable to resistance [23]. This highlights the profound clinical consequences of CRE, particularly in the context of BSIs, which are associated with even higher mortality rates [26].

Furthermore, CPO outbreaks are increasingly being observed in healthcare settings, leading to endemic outbreaks in some regions [26,27]. For example, a prolonged outbreak of NDM-producing *Klebsiella pneumoniae* occurred in Tuscany, Italy, from 2018 to 2021. Genomic sequencing of 117 isolates from 76 patients revealed the spread of a high-risk clone (ST-147), resistant to nearly all antibiotics, highlighting the regional transmission of this multidrug-resistant organism [28]. These infections disproportionately affect critically ill and immunocompromised patients, further deteriorating their clinical outcomes and stressing the urgent need for robust infection control measures, surveillance, and the development of novel therapeutic interventions [25,27].

The substantial mortality rates associated with CRE, particularly in bloodstream infections, underscore the need for effective therapeutic interventions. In response, newer combination therapies, such as β-lactam/β-lactamase inhibitors, have emerged as promising treatment options, demonstrating improved survival rates in clinical studies [25]. However, the growing prevalence of CRE and the phenomenon of heteroresistance, i.e., the presence of antibiotic-resistant subpopulations within a seemingly sensitive population, which may contribute to treatment failure, together form a critical challenge in clinical settings, reinforcing the need for an aligned international response to control the spread of these resistant pathogens [29].

## 3. Epidemiological Methods

### 3.1. Introduction to Epidemiology

Epidemiology is the study of the distribution of diseases and health events within a population, focusing on the factors that influence their prevalence and distribution. This field is essential for informing public health interventions, policy development, and clinical practice aimed at reducing the burden of disease and improving health outcomes. Over time, knowledge gained from epidemiological studies has contributed greatly to fighting infectious diseases, treating chronic diseases, and extending the human lifespan [30].

The integration of advanced data analytics and computational tools in epidemiology has greatly expanded our ability to analyze health data and model disease dynamics. The use of advanced statistical methods, ML algorithms, and big data analytics has improved the identification of risk factors, outbreak monitoring, and evaluation of interventions [31,32]. These innovations will not only provide more accurate predictions, but also open up new research opportunities, potentially revolutionizing public health strategies and improving health outcomes around the world.

### 3.2. Traditional Epidemiological Approaches to Studying AMR

Traditional epidemiological methods have played a key role in understanding the dissemination and impact of AMR. Epidemiological approaches often involve observational studies, such as cohort, case-control, and cross-sectional studies, which help identify risk factors associated with the emergence and distribution of resistance. Surveillance systems, such as the WHO Global Antimicrobial Resistance Surveillance System (GLASS), collect and analyze data on AMR trends in different regions, relying on laboratory data to monitor resistance models [16]. These systems provide critical information to guide public health interventions and antibiotic stewardship programs.

Statistical modeling is another cornerstone of traditional epidemiological methods in AMR research. Regression models, for instance, are commonly used to examine the relationship between antibiotic exposure and the emergence of resistance, adjusting for potential confounding factors. While traditional approaches have provided valuable insights into the epidemiology of AMR, they often rely on certain assumptions and may struggle to capture the complexity and non-linearity of resistance dynamics [31]. Nonetheless, these methods remain fundamental for generating evidence that guides effective public health strategies to combat AMR.

### 3.3. Strengths and Limitations of Epidemiological Approaches in the Context of Rapidly Evolving Resistance Patterns

As in most branches of science, the traditional approach to epidemiology involves the use of frequentist-based statistical approaches that rely on hypothesis testing and computations of probability related to an association or treatment effect. In this area, classic regression models either predict the outcome variable based on a number of other variables or model the relation of individual variables to the outcome [30]. These models are, however, based on certain assumptions; for instance, linearity and the absence of multicollinearity, which present many challenges when the research questions become sophisticated and the amounts of data increase, the so-called “curse of dimensionality” [31].

Despite these challenges, traditional epidemiology has produced successful studies and standardized surveillance systems that have significantly advanced our understanding of AMR. For example, the European Antimicrobial Resistance Surveillance Network (EARS-Net) has been instrumental in tracking resistance trends across Europe, leading to informed public health interventions. The burden of antibiotic-resistant infections in the EU and European Economic Area (EEA) was assessed by another study focusing on cases, deaths, and disability-adjusted life years (DALYs) [33]. Using data from the EARS-Net, the researchers estimated 671,689 infections, with 63.5% linked to healthcare. These infections led to approximately 33,110 deaths and 874,541 DALYs [34]. Similarly, the Global Antimicrobial Resistance Surveillance System (GLASS), launched in 2015, has provided valuable data on AMR patterns worldwide, facilitating global comparisons and targeted responses [16].

However, traditional methods also have limitations, particularly in the context of rapidly evolving resistance patterns. These limitations include the following:

- Data lag. The time required to collect, process, and analyze data can result in delays, making it challenging to respond promptly to emerging resistance threats [35].
- Data completeness. Incomplete data collection and reporting can lead to gaps in understanding the full scope of AMR. Variability in laboratory capacities and surveillance systems across regions further complicates this issue [36].
- Complexity of AMR. AMR is influenced by a multitude of factors, including antibiotic usage, infection control practices, and genetic mechanisms. Traditional methods may struggle to account for these complex, multifactorial influences without integrating more advanced analytical techniques [37].
- Predictive limitations. Traditional epidemiological methods often focus on descriptive and retrospective analyses, which may not be sufficient for predicting future resistance trends or for real-time surveillance [38].

To overcome these challenges, the integration of machine learning and other advanced computational techniques with traditional epidemiological methods is increasingly being advocated. Machine learning can enhance the ability to analyze large and complex datasets, identify hidden patterns, and make real-time predictions, thereby complementing and extending the capabilities of traditional epidemiological approaches [39,40].

## 4. Machine Learning in Healthcare

### 4.1. Introduction to Machine Learning

Machine learning (ML) is a branch of artificial intelligence that involves training algorithms to recognize patterns in data and make predictions or decisions without explicit programming for each task. There are three main types of ML:

- Supervised learning, which involves training an algorithm on a labeled dataset, where the input–output pairs are known. The algorithm learns to map inputs to the correct output. Common algorithms include linear regression, decision trees, and support vector machines [41].
- Unsupervised learning, where the algorithm is trained on data without labeled responses and aims to find hidden patterns or intrinsic structures in the input data. Key techniques include clustering (e.g., k-means and hierarchical clustering) and association (e.g., Apriori algorithm) [42].
- Reinforcement learning, where the algorithm learns by interacting with an environment, receiving rewards or penalties based on the actions it takes. It aims to maximize cumulative rewards over time. Examples include Q-learning and deep reinforcement learning [43].

### 4.2. Key Algorithms and Applications

Machine learning algorithms vary in complexity and application. Some commonly used algorithms for building predictive models to forecast AMR trends, evaluate resistance risk, and provide decision support for treatment planning include the following:

- Linear regression, which is used for predicting a continuous target variable based on one or more predictor variables [44].
- Decision trees, with a flowchart-like structure, where each internal node represents a decision based on an attribute, and each leaf node represents an outcome [45].
- Support vector machine (SVM), which is a classification method that finds the hyperplane that best separates the data into classes [46].
- Neural networks and deep learning models are inspired by the human brain’s structure, capable of learning complex patterns from large datasets, used extensively in image and speech recognition. In the context of deep learning, an artificial neural network with more than one hidden layer is referred to as deep learning, distinguishing it from simpler models with fewer layers [47].

**Figure 1:**
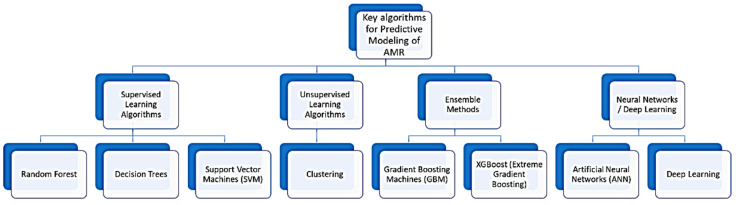
A diagram that outlines some commonly used algorithms in predictive modeling of AMR.

A diagram that outlines some commonly used algorithms in predictive modeling of AMR is shown in Figure 1.

### 4.3. Bridging Terminology: Aligning Epidemiology and Machine Learning Concepts

In the fields of epidemiology and ML, certain terminologies and concepts align closely, despite their usage in different contexts. In epidemiology and biostatistics, terms such as dependent variable, outcome variable, and response variable are used to refer to the variable that is being measured or predicted. In ML and statistical modeling, this concept corresponds to the label or class that the model aims to predict. Conversely, independent variables, predictor variables, and explanatory variables in epidemiology are equivalent to features in ML, which are the inputs or attributes used to predict the label [31].

A common tool in epidemiological studies is the contingency table or 2 × 2 table, which displays the relationship between two categorical variables. In ML, this is referred to as a confusion matrix, which is used to evaluate the performance of classification models by showing the actual versus predicted classifications. Sensitivity in epidemiology, also known as recall in ML, measures the true-positive rate of a test. The positive predictive value in epidemiology, which assesses how many of the positive test results are true positives, is analogous to precision in ML [31].

When discussing outcome groups, the majority class in ML represents the outcome group with the highest frequency, while the minority class refers to the outcome group with the lowest frequency. In epidemiology, this concept is reflected in the proportion of cases in each category of the outcome variable, particularly when the outcome is categorical. This is similar to the concept of class balance in machine learning, which denotes the distribution of cases among different classes [31]. Understanding these terms and their equivalents across both fields improves clarity and facilitates effective communication when merging epidemiological insights in ML methods.

### 4.4. Benefits of Machine Learning in Analyzing Complex Biological Data and Predicting Trends

Machine learning offers numerous benefits in healthcare, especially in analyzing complex biological data and predicting trends. One significant advantage is its ability to handle big data. ML algorithms can process and analyze vast amounts of information from various sources, such as electronic health records (EHRs), genomic data, and medical imaging [48]. Additionally, ML models excel in predictive analytics by identifying patterns and correlations in historical data, enabling the prediction of disease outbreaks, patient outcomes, and treatment responses [49].

Another key benefit is in personalized medicine, where ML helps tailor medical treatment to individual patients based on their genetic profile, lifestyle, and other factors, thereby improving treatment efficacy and reducing adverse effects [40]. Furthermore, advanced ML models significantly enhance diagnostics, offering high accuracy in diagnosing diseases from medical images, pathology slides, and other diagnostic tests [50,51].

## 5. Integration of Machine Learning and Epidemiology

Integrating ML with epidemiological data enhances the ability to predict and respond to resistance trends. This integration involves handling diverse data sources, developing predictive models, and conducting real-time surveillance.

### 5.1. Data Sources and Preprocessing Techniques

Data sources: Both machine learning and epidemiology rely on several data sources to offer good models and meaningful outputs. Some of the main categories these data sources fall under include the following:

- Genomic data. Genomic sequences, which comprise DNA or RNA of both pathogens and hosts, are helpful in the identification of genetic markers responsible for specific traits, such as drug resistance and virulence, which become indispensable for full comprehension of infectious disease mechanisms and epidemiology [52].
- Clinical data. Patients’ electronic health records (EHRs) are a rich source of vital information, such as demographics, diagnosis, treatment, and results/outcomes. This dataset captures detailed patient histories that can be used to track disease progression and treatment responses [53].
- Environmental data. Environmental factors, such as air quality, water quality, and climatic variables, may affect dissemination of infectious diseases. Such information may even indicate changing environmental conditions and, thus, the impact on disease transmission [54].
- Sociodemographic data. Information on aspects such as the population’s economic status, density, and education level is critical for understanding disease transmission within populations. More so, such elements can bring to light health-related disparities and susceptibilities [55].

**Figure 2:**
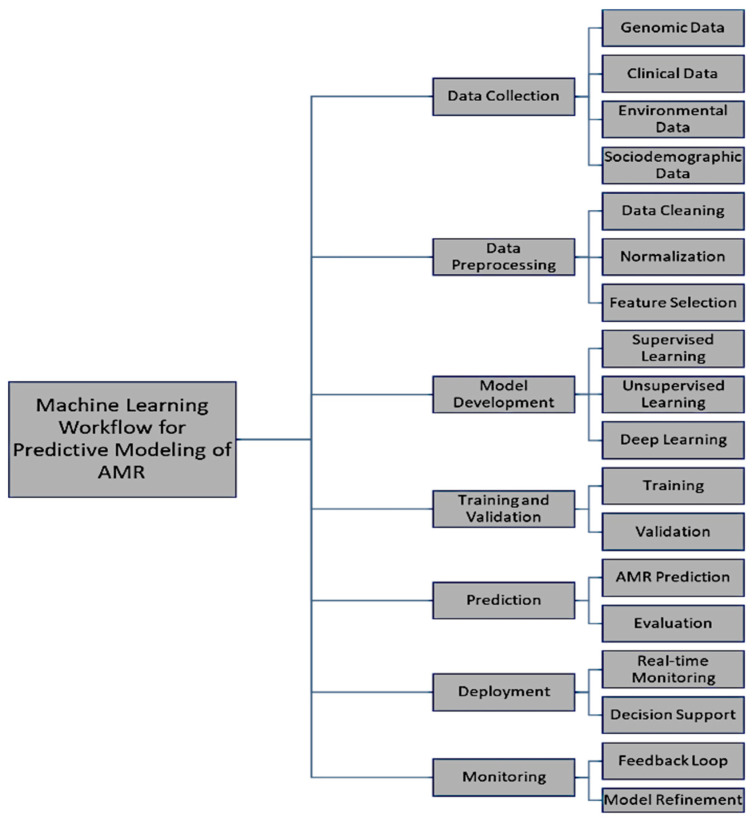
Machine learning workflow for predictive modeling of AMR.

Preprocessing techniques for data preparation: To ensure the accuracy and usability of these diverse datasets, several preprocessing steps are critical, as follows:

- Data cleaning. Identification and correction of errors, inconsistencies, or incompleteness. Cleaning the data assures dependability and quality within the data and, hence, validation of ML models [56].
- Normalization. When several datasets are combined, normalization is necessary to standardize their scale. Certain algorithms are sensitive to the range of the data; thus, normalization ensures that no feature dominates the model because of a difference in its scale [57].
- Feature selection, which determines the most relevant variables. This, in turn, aids model performance by reducing dimensionality and weeding out insignificant or redundant data. It is totally focused on the most important part of the data and yields a higher performance with low computational complexity [58]. Figure 2 outlines the steps for a machine learning workflow for predictive modeling of AMR. This workflow illustrates how machine learning models handle diverse datasets for predicting AMR trends and providing clinical decision support.

Challenges and solutions in data integration: Integrating ML with epidemiological data allows for more precise modeling of disease dynamics and resistance patterns. For example, linking genomic data with clinical outcomes can reveal genetic determinants of drug resistance, while environmental and sociodemographic data provide a broader context within public health frameworks [59,60,61]. Genomic data, in particular, have become an important component of epidemiological analysis, especially in understanding the molecular mechanisms behind resistance and disease transmission. The integration with traditional epidemiological approaches has transformed how infectious diseases, such as carbapenem resistance, are studied [62].

However, combining these diverse data sources poses several challenges. A key issue is data format inconsistencies, as genomic, clinical, and environmental datasets often use different formats, complicating integration. Standardizing formats can help streamline the integration process and improve the efficiency of analysis.

Another challenge is data privacy and security, particularly when dealing with sensitive clinical and sociodemographic information. Additionally, missing data present a hurdle for comprehensive analysis. All these challenges and possible solutions are discussed in Section 7. 

### 5.2. Predictive Modeling

Development of predictive models: ML has become a powerful tool in the fight against carbapenem resistance by enabling the development of sophisticated predictive models. These models utilize various ML techniques to analyze large and complex datasets, aiming to forecast resistance patterns and guide effective interventions [63,64,65]. Supervised learning algorithms are commonly employed, where models are trained on historical data that include information on infection cases, antibiotic usage, and resistance outcomes [66]. This training helps the models identify patterns and correlations that indicate the likelihood of resistance [67,68].

Key features incorporated into these predictive models include patient demographics, clinical history, hospital environment, and the genetic characteristics of pathogens [69]. By considering these variables, ML models provide valuable insights into the emergence and spread of carbapenem-resistant infections. For example, models can predict how changes in antibiotic prescribing practices or hospital infection control measures might impact resistance trends [70]. This proactive approach allows healthcare providers to implement timely and targeted interventions to manage and mitigate the spread of resistance [71,72,73]. 

Evaluation metrics for model performance: The performance of predictive models is evaluated using various metrics to ensure their accuracy and reliability. Common evaluation metrics include the following:

- Accuracy. The proportion of true results (both true positives and true negatives) among the total number of cases examined. It indicates the overall correctness of the model [74].
- Precision. The proportion of true-positive results among all positive results predicted by the model. It measures the model’s ability to correctly identify true resistance cases without including false positives [75].
- Recall (sensitivity). The proportion of true-positive results among all actual positive cases. It assesses the model’s ability to detect true resistance cases [76].
- F1 score. The harmonic mean of precision and recall, providing a single metric that balances both. It is particularly useful when the data are imbalanced, meaning the number of positive cases is much smaller than the number of negative cases [77].
- Area under the receiver operating characteristic (ROC) curve (AUROC). A plot of the true-positive rate against the false-positive rate at various threshold settings. The AUROC provides a single measure of the model’s ability to discriminate between positive and negative cases [78]. In the context of predicting antibiotic resistance, it measures how effectively the model can differentiate between cases where bacteria are resistant to an antibiotic and cases where they are not.

Case studies demonstrating successful predictions and early detection of resistance trends: Several case studies exemplify the successful application of ML in predicting and managing carbapenem resistance. Machine learning has proven highly effective in predicting BSIs and detecting antimicrobial resistance trends, particularly for carbapenem-resistant Gram-negative bacteria (CRGNB) in intensive care unit (ICU) patients. In a multicenter study from China, a random forest algorithm achieved an AUROC of 0.88 for CRGNB prediction and 0.86 for overall BSI prediction, enabling early intervention and targeted antibiotic therapies [63]. Similarly, a 2022 study demonstrated that ML algorithms, particularly random forest, could predict CRGNB carriage with 85.92% accuracy [79]. These models integrate variables, such as prior antibiotic use, mechanical ventilation, and invasive procedures, to provide real-time monitoring of resistance patterns. The early detection of CRGNB infections allows hospitals to optimize antimicrobial stewardship, reduce unnecessary use of broad-spectrum antibiotics, and better target high-risk patients, significantly improving clinical outcomes and infection control efforts.

Another study focused on developing and validating a ML-based algorithm to predict CR bacterial infections at the time of culture collection, achieving a sensitivity of 30%, a positive predictive value (PPV) of 30%, and a negative predictive value (NPV) of 99%, with *Pseudomonas* species accounting for 58% of the resistant infections. Integration of the model into the EHR system could enable real-time predictions, improving antibiotic stewardship by allowing early intervention and reducing unnecessary use of last-resort antibiotics [80]. 

Despite limitations, including reliance on single-center datasets, in most of the studies, these models showed promise for broader application, particularly in high-risk healthcare settings, as they can be easily retrained with additional data to reflect changing microbiological trends. This early detection is crucial for improving antimicrobial stewardship, reducing the unnecessary use of broad-spectrum antibiotics, and focusing treatments on high-risk patient groups. As these tools evolve, they promise to further optimize infection control strategies and enhance patient outcomes in hospital settings [81].

### 5.3. Epidemiological Insights

Enhancing traditional epidemiological analysis with machine learning: Machine learning (ML) significantly enhances traditional epidemiological analysis by providing advanced tools for data processing, pattern recognition, and predictive modeling [31,82]. Traditional epidemiology often relies on statistical methods that may not fully capture complex interactions within large datasets [31]. In contrast, ML algorithms can handle high-dimensional data, identify non-linear relationships, and uncover hidden patterns that are not apparent through conventional methods. This capability allows for more accurate risk assessments and targeted interventions.

Identifying risk factors and transmission patterns: ML models can integrate diverse data sources, such as genomic, clinical, environmental, and sociodemographic data, to identify risk factors and transmission patterns of AMR. For example, ML algorithms can analyze patient records to determine the factors associated with higher risks of infection with antibiotic-resistant pathogens. By mapping these factors, ML helps in understanding how resistance spreads within communities and healthcare settings [71,83,84,85].

Real-time surveillance and outbreak prediction: Real-time surveillance and outbreak prediction have become increasingly effective through the application of ML algorithms [86,87]. These algorithms excel at continuously analyzing incoming data to detect early signs of an outbreak, providing timely alerts to public health authorities and enabling them to take immediate action [88,89]. Predictive models can forecast the spread of antimicrobial resistance based on current trends, allowing for proactive measures to mitigate the impact of potential outbreaks. ML-driven surveillance systems, in particular, offer significant advantages in monitoring hospital data for unusual antibiotic resistance patterns, facilitating rapid responses to emerging threats [90,91]. For instance, a study by Caglayan developed a predictive framework using ML to identify ICU patients at risk of colonization with multi-drug-resistant organisms, including CRE [92]. The analysis of 4670 ICU admissions showed that the best-performing model achieved 82% sensitivity and 83% specificity. Among the key risk factors identified were prior stays in long-term care facilities and recent isolation procedures. This tool can be instrumental for clinicians in implementing timely infection control measures for high-risk patients, ultimately improving patient outcomes and preventing the spread of resistant infections.

### 5.4. Real-World Applications

Examples of machine learning in hospitals and public health: Machine learning has made notable strides in improving health outcomes by analyzing large-scale data to predict AMR trends. For instance, ML models have been applied to monitor and predict outbreaks of multidrug-resistant tuberculosis (MDR-TB). By integrating diverse data sources, such as patient records, radiomic features (such as cavitation), and sociodemographic information, ML enhances the ability to track and control the spread of MDR-TB more effectively than traditional methods [93,94]. This approach allows public health officials to allocate resources more efficiently, focusing on high-risk areas and implementing targeted interventions to curb resistance. Such predictive modeling empowers public health systems to act preemptively, potentially reducing the overall burden of drug-resistant infections.

Success stories and lessons learned from integrating machine learning with epidemiology: There are several success stories where ML integration with traditional epidemiological tools has led to significant improvements in patient care and infection control. For instance, in one large healthcare system, ML algorithms outperformed traditional scoring systems, such as the Modified Early Warning Score (MEWS), Sequential Organ Failure Assessment (SOFA), and Systemic Inflammatory Response Syndrome (SIRS), in predicting severe sepsis. The ML model used only patient age and six vital signs from electronic health records (EHRs) to enhance early sepsis detection, while also reducing alarm fatigue, a prevalent concern in patient safety [95]. This demonstrates the ability of ML to provide more precise alerts, improving both detection and clinical workflow.

Similarly, the InSight algorithm, developed at the University of California, San Francisco Medical Center, achieved impressive results in detecting sepsis and septic shock. It reached an area under the receiver operating characteristic curve (AUROC) of 0.92 for sepsis detection and 0.96 for predicting septic shock four hours before onset [96]. This ML model not only outperformed existing sepsis scoring systems but also proved to be robust in the face of missing data, adaptable across institutions through transfer learning, and generalizable to various clinical settings. Its strong performance underscores the potential of ML to drive improvements in early diagnosis and intervention, which are critical in conditions such as sepsis, where early treatment significantly improves outcomes.

Machine learning has also contributed to better antibiotic stewardship programs. One study demonstrated the effectiveness of the XGBoost algorithm (https://xgboost.readthedocs.io/en/latest/index.html, accessed on 19 August 2024) in predicting antibiotic resistance for three Gram-negative bacteria: *Escherichia coli*, *Klebsiella pneumoniae*, and *Pseudomonas aeruginosa*. Using data from 15,695 hospital admissions in the UK, the ML model slightly outperformed clinicians in selecting appropriate antibiotics, achieving an AUROC of 0.70 [97]. Importantly, this approach could reduce the use of broad-spectrum antibiotics by up to 40%, a key step in combating the development of further antibiotic resistance. Despite these promising results, the study called for further validation through prospective trials to ensure its effectiveness and acceptance in clinical practice.

Another notable example of ML-driven antibiotic stewardship is the successful reduction of extended-spectrum beta-lactamase (ESBL)-targeted therapies in hospital settings. An ML program identified patients at low risk for ESBL-producing pathogens, allowing for more targeted use of antibiotics instead of relying on broad empirical carbapenem use [72]. This precision-guided treatment approach not only helps preserve the efficacy of carbapenems but also reduces the risk of fostering additional resistance.

The lessons learned from these implementations emphasize several key points for integrating ML into healthcare. First, high-quality data are essential for accurate and reliable predictions. Second, interdisciplinary collaboration between data scientists, healthcare professionals, and public health officials is critical to ensure that ML models are not only computationally robust but also clinically relevant. Third, continuous evaluation and refinement of ML models are necessary to maintain their effectiveness, especially as healthcare environments and microbial landscapes evolve. Effective communication between all stakeholders ensures that the full potential of ML can be realized in improving patient outcomes and public health [98].

### 5.5. Case Studies

Specific instances where machine learning and epidemiology have been used to address carbapenem resistance: Several case studies highlight the successful integration of ML and epidemiological methods to combat carbapenem resistance. One notable example is a study conducted in a large urban hospital, where ML algorithms were used to predict the occurrence of carbapenem-resistant *Klebsiella pneumoniae* (CRKP) infections. The predictive model analyzed patient data, including demographics, medical history, and previous antibiotic use, to identify individuals at high risk of developing CRKP infections [99]. This approach allowed for early intervention and targeted infection control measures, significantly reducing the incidence of CRKP infections. Another case study involved the use of ML to analyze national surveillance data on CRIs in the United States [80]. A machine learning model was developed to predict CRIs using data from 68,472 patients. Built with extreme gradient boosting, the model achieved an AUC of 0.846, with a 99% negative predictive value. Despite moderate sensitivity, it effectively ruled out CR infections, aiding in early detection and intervention in healthcare settings.

Impact on patient outcomes and public health interventions: The integration of ML and epidemiology offers significant theoretical advantages, such as enhancing patient outcomes and public health interventions. In the hospital setting, predictive models could enable healthcare providers to implement timely and appropriate infection control measures, potentially reducing the spread of carbapenem-resistant pathogens and improving patient outcomes [92]. On a broader scale, ML-driven epidemiological studies have the potential to significantly influence public health policies and resource allocation. By accurately predicting areas at high risk for carbapenem resistance, it is postulated that public health authorities can more effectively prioritize interventions, such as enhanced surveillance, targeted education campaigns, and stricter antibiotic stewardship programs [100,101]. This targeted approach could lead to a notable reduction in the incidence of carbapenem-resistant infections and a subsequent improvement in overall public health outcomes [102]. A multinational cohort study by Giannella et al. developed a risk prediction model for CRE infections following liver transplantation [90]. The model identifies several risk factors, including prior antibiotic use, specific comorbidities, and healthcare exposure, providing clinicians with a valuable tool to predict CRE infections and implement preventive measures in high-risk patients. Freire et al. extended this work by proposing a predictive risk score for CRE colonization prior to liver transplantation, using clinical and epidemiological data [91]. This risk score could guide preventive measures, such as targeted antibiotic prophylaxis, to address the challenge of identifying CRE colonization in patients on the waiting list. Public health authorities can use these findings to prioritize resources, focusing efforts on hospitals with higher incidences of CRE carriage among vulnerable populations, thereby reducing overall healthcare costs and improving patient safety. 

The following table (Table 1) summarizes prediction models for carbapenem resistance, highlighting data sources, accuracy, and ML algorithms used. These studies illustrate how combining ML techniques with epidemiological data enhances early detection and intervention for CRIs.

## 6. Challenges and Future Directions

### 6.1. Technical and Ethical Challenges

Data quality and completeness: One of the primary technical challenges in integrating ML with epidemiological studies is ensuring the quality and completeness of the data. Addressing data quality is crucial, as accurate, comprehensive datasets are fundamental to reliable modeling and outcomes. Inconsistent data collection methods, missing values, and errors in data entry can lead to biased models and inaccurate conclusions. This challenge is amplified when integrating data from diverse sources, such as genomic, clinical, and environmental datasets, which often suffer from data format inconsistencies. Standardizing data collection protocols and formats—using systems such as HL7 for clinical data—can streamline integration and improve data quality [103]. Robust data cleaning, validation processes, and advanced imputation techniques, such as k-nearest neighbors (KNN) or multiple imputation by chained equations (MICE), are crucial for addressing these issues and ensuring the reliability of the datasets. [104].

Interpretability of machine learning models: ML models, especially deep learning algorithms, often deliver high accuracy but lack interpretability, which limits their adoption in clinical and epidemiological settings. Healthcare professionals need to trust and understand how decisions are made. This “black box” nature becomes a barrier, particularly when integrated with complex epidemiological data, such as genomic or environmental information. Developing interpretable models and techniques to explain predictions can help build trust among healthcare providers and epidemiologists, ensuring that decisions are transparent and justifiable [105].

Ethical considerations in data use and patient privacy: Ethical concerns about patient privacy and data security are frequently raised in relation to large datasets in ML. The integration of clinical and sociodemographic data further complicates these matters, as sensitive patient information must be securely stored and anonymized. Strict compliance with data protection regulations, such as the General Data Protection Regulation (GDPR) and Health Insurance Portability and Accountability Act (HIPAA), is essential, as are robust encryption techniques to safeguard data. 

The GDPR sets strict rules for the processing of sensitive patient data, including medical records, and requires that personal data be anonymized or pseudonymized before transfer. This is essential to ensure patient confidentiality and prevent reidentification. However, these requirements can limit the availability of detailed data, which are essential for effective ML models in AMR monitoring. For example, while anonymization can protect individual privacy, it reduces the granularity of the data, which can impact the ability of models to detect specific AMR trends or patterns.

Additionally, the GDPR places limitations on the transfer of personal data outside the European Economic Area (EEA). In the case of AMR monitoring, which often requires international cooperation, these regulations may prevent data sharing with countries that do not have equivalent data protection laws. Ensuring compliance requires setting up complex legal frameworks, such as standard contractual clauses, which can slow down data exchange and collaboration efforts.

Furthermore, the integration of clinical, genomic, and sociodemographic data for AMR surveillance raises ethical issues regarding consent and transparency [106]. Under the GDPR, patients must explicitly consent to the use of their data and have the right to withdraw this consent. Balancing these ethical considerations with the need for large, diverse datasets to improve predictive modeling for AMR poses a significant challenge.

Addressing biases in data and algorithms: Bias in data and algorithms poses a significant challenge, leading to unfair or discriminatory outcomes. Biases may originate at various stages, from data collection to model training and deployment. For example, if a training dataset is not representative of the broader population, models may perform poorly for underrepresented groups, resulting in inequitable healthcare delivery. When integrating diverse datasets, such as genomic, clinical, environmental, and sociodemographic data, it is crucial to monitor biases continuously. Algorithms should be developed with fairness in mind, ensuring equitable outcomes across all population groups [107].

Model generalizability in diverse healthcare contexts: The generalizability of machine learning models remains a significant challenge, particularly in healthcare settings with varying resources, patient populations, and infrastructure [108]. Factors contributing to CR may differ widely across regions and environments, further complicating the application of predictive models across diverse contexts. The variability in EHRs and laboratory data from different institutions or countries adds to the complexity, as these data may not be easily reproducible or standardized. To address this, it is crucial to tailor models to specific local contexts and continuously refine them with locally sourced data to ensure that predictions remain accurate and effective. Even when algorithms are developed, their prospective validation in diverse clinical environments is essential to ensure generalizability and reliability across various healthcare systems [105].

Addressing the limitations and operational challenges of machine learning in clinical implementation: While the integration of ML into healthcare holds great promise for enhancing patient outcomes and streamlining public health initiatives, it is crucial to acknowledge the limitations and operational challenges that accompany its implementation. One significant barrier is the requirement for large, high-quality datasets, which can be difficult to obtain, especially in settings with limited data-sharing practices [106]. Furthermore, integrating ML into existing clinical workflows may encounter resistance from staff due to concerns about reliability and accountability, as well as the need for comprehensive training on new technologies. Addressing these challenges through targeted strategies and ongoing evaluation will be essential for realizing the full potential of ML in improving healthcare delivery [107].

### 6.2. Future Directions

Potential advancements in machine learning algorithms and computational power: Advancements in ML algorithms and computational power are poised to significantly enhance the capabilities of epidemiological studies. The development of more sophisticated algorithms, such as deep learning models with improved architectures, can lead to better performance in detecting and predicting patterns of AMR [109]. Additionally, the increasing availability of high-performance computing resources, including cloud-based platforms and specialized hardware, such as GPUs and TPUs, allows for the processing of larger datasets and the training of more complex models [110].

Opportunities for integrating other emerging technologies (e.g., AI and big data analytics) with epidemiology: The integration of other emerging technologies with epidemiology presents numerous opportunities for advancing the field. Artificial intelligence (AI), big data analytics, and the Internet of Things (IoT) can provide valuable insights into the spread and control of AMR. For example, AI can enhance the analysis of genomic data to identify resistance genes, while big data analytics can uncover trends and correlations in vast datasets from various sources, including electronic health records and environmental sensors [111]. The IoT can facilitate real-time monitoring of environmental conditions and the spread of infectious diseases, enabling more timely and effective public health interventions [112].

Recommendations for policy and practice to maximize the impact of these interdisciplinary approaches: To maximize the impact of interdisciplinary approaches combining ML and epidemiology, several key recommendations should be implemented, as follows:

- Standardization of data collection. The establishment of standardized protocols for data collection and reporting is critical. This will enhance the quality and comparability of datasets, which are vital for the effectiveness of ML models in epidemiological studies [113].
- Investment in infrastructure. Governments and organizations should invest in the necessary infrastructure, including high-performance computing resources and secure data storage solutions, to support the integration of ML and epidemiological methods [114].
- Interdisciplinary collaboration. It is essential to foster collaboration among data scientists, epidemiologists, healthcare professionals, and policymakers. Such interdisciplinary partnerships can drive the creation of effective and practical solutions for tackling antimicrobial resistance (AMR) and other public health challenges [40].
- Ethical and regulatory frameworks. Developing comprehensive ethical and regulatory frameworks is crucial. These frameworks should address privacy concerns, data security, and the responsible use of AI and ML technologies, thereby ensuring public trust and the successful deployment of these approaches in real-world settings [115].

### 6.3. The Role of Interdisciplinary Collaboration in Advancing This Field 

Interdisciplinary collaboration plays a crucial role in advancing the integration of ML and epidemiology. By bringing together experts from various fields, including computer science, public health, medicine, and social sciences, collaborative efforts can leverage diverse perspectives and expertise to tackle complex challenges associated with AMR. Such collaborations can lead to the development of innovative models, the identification of novel intervention strategies, and the creation of comprehensive public health policies that are informed by data-driven insights [116]. 

In essence, the integration of ML and epidemiology is not just about combining tools from different disciplines, but about creating a synergistic framework where each discipline informs and enhances the other. This collaborative approach can lead to breakthroughs in understanding and combating AMR, ultimately leading to more effective interventions, better patient outcomes, and stronger public health systems. By leveraging the strengths of various fields, interdisciplinary collaboration can drive the innovation needed to address one of the most pressing global health challenges of our time.

## 7. Conclusions

The integration of ML and epidemiology has the potential to transform public health interventions by offering precise predictions and tailored approaches to infection control. ML-driven models help healthcare systems and public health authorities respond more effectively to the growing challenge of AMR, reducing the spread of resistant infections, improving patient outcomes, and optimizing the use of resources. This data-driven approach promises to significantly enhance both patient care and public health efforts.

Looking ahead, the future of combining ML and epidemiology holds great promise for combating AMR. As ML algorithms and computational power continue to advance, their application in healthcare will become increasingly sophisticated and impactful. The integration of emerging technologies, such as AI and big data analytics, with epidemiological methods will further enhance the ability to predict, monitor, and respond to resistance trends. This synergy will enable more efficient resource allocation, early detection of resistance patterns, and timely interventions, ultimately helping to curb the growing threat of antimicrobial resistance on a global scale.

Furthermore, the broader impact of this integration will foster a more proactive and informed approach to public health, enabling healthcare systems to implement targeted interventions and policies that not only address current challenges but also anticipate future outbreaks of AMR. By leveraging the power of ML in epidemiology, we can build a more resilient and responsive healthcare infrastructure that significantly improves global health outcomes.

## Figures and Tables

**Table 1:** Summary of prediction models for carbapenem resistance: integration of machine learning and epidemiological data across studies.

No.	Author	Geographical Setting	Publication Year	Medical Setting	Data Source	ML Algorithms	Performance Evaluation	Bacterial Species
1	Timothy Sullivan [99]	United States (Single Center)	2018	Hospital setting	EHR data, *Klebsiella pneumoniae* bacteremia cases	Multiple logistic regression	AUROC: 0.731, Sensitivity: 73%, Specificity: 59%, PPV: 16%, NPV: 95%	*Klebsiella pneumoniae* (Carbapenem-resistant)
2	Ariane Khaledi [71]	Germany, Spain	2020	Clinical settings, multicenter	Whole genome sequencing (WGS), transcriptomic data, gene presence/absence, expression profiles	Machine Learning (unspecified classifiers)	Sensitivity: 0.8–0.9, Predictive values: >0.9	*Pseudomonas aeruginosa* (Carbapenem-resistant)
3	Ed Moran [97]	United Kingdom (Single Center)	2020	Hospital setting	Blood and urine cultures, demographics, microbiology and prescribing data	XGBoost	AUROC: 0.70, Point-scoring tools: AUROC 0.61 to 0.67, estimated reduction in broad-spectrum antibiotic use by 40%	*Escherichia coli*, *Klebsiella pneumoniae*, *Pseudomonas aeruginosa*
4	Ryan J. McGuire [80]	United States (Single Center)	2021	Tertiary-care academic medical center	Demographics, medications, vital signs, procedures, lab results, cultures	Extreme gradient boosting (XGBoost)	AUROC: 0.846, Sensitivity: 30%, PPV: 30%, NPV: 99%	Carbapenem-resistant bacteria
5	Maddalena Giannella [90]	Multinational	2021	Liver transplantation units (multicenter)	Demographics, clinical data, mechanical ventilation, acute renal injury, surgical reintervention	Multivariable logistic regression, Fine-Gray subdistribution hazard model	AUROC: 74.6 (derivation), AUROC: 73.9 (bootstrapped validation), Brier Index: 16.6	Carbapenem-resistant Enterobacteriaceae (CRE)
6	Qiqiang Liang [79]	China (Single Center)	2022	Intensive care unit (ICU)	Demographics, screening records, clinical data, vitals	Random forest, XGBoost, decision tree, logistic regression	AUROC: 0.91 (random forest), 0.89 (XGBoost, decision tree), 0.78 (logistic regression)	Carbapenem-resistant Gram-negative bacteria (CRGNB)
7	Maristela Pinheiro Freire [91]	Brazil, Italy	2022	Liver transplantation units (multicenter)	Antibiotic use, hepato-renal syndrome, CLIF-SOFA scores, cirrhosis complications	Machine learning (unspecified)	Sensitivity: 66%, Specificity: 83%, NPV: 97%	Carbapenem-resistant Enterobacterales (CRE)
8	Çaǧlar Çaǧlayan [92]	United States (Single Center)	2022	Intensive care unit (ICU)	EHR, MDRO screening program, sociodemographic and clinical factors	Logistic regression (LR), random forest (RF), XGBoost	Sensitivity: VRE 80%, CRE 73%, MRSA 76%, MDRO 82%; Specificity: VRE 66%, CRE 77%, MRSA 59%, MDRO 83%	MRSA, VRE, Carbapenem-resistant Enterobacteriaceae (CRE)
9	Qiqiang Liang [63]	China (Single Center)	2024	Intensive care unit (ICU)	Demographics, mechanical ventilation, invasive catheterization, carbapenem use history	Random forest, XGBoost, SVM	AUROC: random forest 0.86, XGBoost (infection): 0.86, SVM: 0.88, RF (CRGNB): 0.87	Carbapenem-resistant Gram-negative bacteria (CRGNB)
10	Yun Li [65]	China/USA	2024	Intensive care unit (ICU)	Electronic health record data (PLAGH-ICU, MIMIC-IV)	Machine learning models	AUROC: 0.786 (PLAGH-ICU), 0.744 (MIMIC-IV)	Multidrug-resistant organisms (MDRO), including carbapenem-resistant species
11	Bing Liu [64]	China (Single Center)	2024	Multiple hospital settings	Whole-genome sequencing (WGS) data, metagenomic sequencing (MGS), genomic features	Machine learning (unspecified algorithms)	AUROC: 0.906 (IPM), 0.925 (MEM), PPV: 0.897 (IPM), 0.889 (MEM)	*Pseudomonas aeruginosa* (Carbapenem-resistant)

AUROC: area under the receiver operating characteristic curve; CLIF-SOFA: Chronic Liver Failure–Sequential Organ Failure Assessment; EHR: electronic health record; SVM: support vector machine; LR: logistic regression; RF: random forest; XGBoost: extreme gradient boosting; CRE: carbapenem-resistant Enterobacteriaceae; MRSA: methicillin-resistant *Staphylococcus aureus*; VRE: vancomycin-resistant Enterococci; MDRO: multidrug-resistant organisms; NPV: negative predictive value; PPV: positive predictive value; CRGNB: carbapenem-resistant Gram-negative bacteria; PLAGH-ICU: a Chinese hospital ICU dataset; MIMIC-IV: Medical Information Mart for Intensive Care IV; IPM: imipenem; MEM: meropenem; WGS: whole-genome sequencing; MGS: metagenomic sequencing.

## Data Availability

Not applicable.
